# Environmentally vulnerable noble chafers exhibit unusual pheromone-mediated behaviour

**DOI:** 10.1371/journal.pone.0206526

**Published:** 2018-11-01

**Authors:** Deborah J. Harvey, József Vuts, Antony Hooper, Paul Finch, Christine M. Woodcock, John C. Caulfield, Marcin Kadej, Adrian Smolis, David M. Withall, Sarah Henshall, John A. Pickett, Alan C. Gange, Michael A. Birkett

**Affiliations:** 1 School of Biological Sciences, University of London, Egham, Surrey, United Kingdom; 2 Department of Biointeractions and Crop Protection, Rothamsted Research, Harpenden, Herts, United Kingdom; 3 School of Biological and Chemical Sciences, Queen Mary University of London, London, United Kingdom; 4 Department of Invertebrate Biology, Evolution and Conservation, Institute of Environmental Biology, Faculty of Biological Science, University of Wrocław, Wrocław, Poland; 5 School of Chemistry, Cardiff University, Cardiff, Wales, United Kingdom; 6 Buglife–The Invertebrate Conservation Trust, Orton Waterville, Peterborough, United Kingdom; University of California-Davis, UNITED STATES

## Abstract

Conserving populations of environmentally vulnerable insect species requires a greater understanding of the factors that determine their abundance and distribution, which requires detailed knowledge of their population and community ecology. Chemical ecological tools such as pheromones can be used for non-destructive monitoring of scarab beetle populations, enabling European countries to detect and, in some cases, map the range of some of these species, proving a valuable technique for monitoring elusive saproxylic beetles. In this paper, we investigated the behavioural and chemical ecology of the noble chafer, *Gnorimus nobilis* L., a model insect species of conservation concern across a Europe-wide distribution, and a red-listed UK Biodiversity Action Plan priority species. We identified a potential pheromone of adult beetles using electrophysiological recordings, behavioural measurements and field trials in the UK. *Gnorimus nobilis* is highly unusual in that although both sexes produce, at high metabolic cost, the natural product 2-propyl (*E*)-3-hexenoate, it only attracts males. This pattern of chemical signalling makes the classification of the compound, based on current semiochemical terminology, somewhat problematic, but in our view, it should be termed an aggregation pheromone as a consequence of the production pattern. Since both sexes emit it, but apparently only males respond positively to it, 2-propyl (*E*)-3-hexenoate may reflect an intermediate evolutionary stage towards developing into a sex-specific signal. From an applied perspective, our study provides a model for the non-invasive surveillance of cryptic vulnerable insect species, without the need for habitat searching or disturbance, and continuous human monitoring.

## Introduction

Forests are a key resource of biological diversity, with their structural complexity providing ideal habitats for a particularly rich array of wildlife. Within forests, decaying wood is a key indicator for biodiversity and the species conservation value of a forest, and is an important substrate for a wide variety of insect species [1]. Whilst the harvesting of wood from forests across the UK and Europe is largely sustainable, levels of dead and decaying wood in most European countries remain below the optimum from the perspective of preserving biodiversity [2]. As a consequence, several insect species that utilize dead and decaying wood for their habitat are considered as environmentally vulnerable [3]. Some saproxylic beetles, such as those in the Scarabidaeae, have specific environmental requirements, i.e., they can develop on accumulating heartwood mould in the base of hollowing trunks, a process that is primarily driven by wood-decaying fungi [4]. Across Europe, this environment supports a unique variety of rare saproxylic communities. However, the European Red List of saproxylic beetles states that 11% of species are threatened, and the populations of a further 14% are in decline [5]. Perhaps worryingly, more than half of the species are classified as data-deficient, i.e., there is insufficient knowledge to assess their risks of extinction. Furthermore, despite their role in the ecosystem as decomposers and nutrient recyclers, few countries have any systematic monitoring schemes for saproxylic beetles and, with the exception of the stag beetle, *Lucanus cervus* L. (Coleoptera: Lucanidae) [6,7], there are no European initiatives to coordinate successful monitoring regimes to improve this situation.

To conserve populations of environmentally vulnerable insect species, including saproxylic beetles, a greater understanding of the factors that determine abundance, distribution and specialisation is urgently required, which in turn necessitates more detailed knowledge of their population, community and evolutionary ecology. In this paper, we report on understanding the behavioural and chemical ecology of the noble chafer, *Gnorimus nobilis* L. (Coleoptera: Scarabaeidae), a model insect species of conservation concern across Europe, and which is a red-listed UK Biodiversity Action Plan priority species [8]. *Gnorimus nobilis* is considered to be a ‘remnant of the saproxylic beetle assemblage of Britain’s post-glacial wildwood, which has survived through a history of woodland pasture management systems in certain refugia’, and thus an indicator of sites with high level of ecological continuity [9]. Adult *G*. *nobilis* are iridescent green and the larvae are found largely in brown rot in the cavities of broadleaf trees in deciduous forests in mainland Europe [10], while in the UK, it is mainly confined to veteran orchards (predominantly *Prunus*), with a few records from one forested area [5,11]. The latter suggests that the distribution of *G*. *nobilis* may be wider than habitat searching has revealed, possibly since once a habitat is identified as suitable, efforts to determine presence are focussed on other regions with a similar habitat profile. The adults are diurnal and can be occasionally seen feeding and mating on flowers of Apiaceae and Adoxaceae, e.g., *Heracleum sphondylium* L. and *Sambucus nigra* L., respectively [10,11]. Currently, the method for detecting *G*. *nobilis* presence relies on finding larval frass within tree hollows, since adults are rarely seen and only in very low numbers. The frass is lozenge-shaped and approximately 3 mm long, distinguishing it from the frass of other co-occurring species [11]. However, the larval habitat tends to be dry and above the ground, meaning that the decay time is longer than the two-year larval stage. Thus, this method leads to population numbers being overestimated. Confirming the presence of larvae within a cavity containing frass requires manually searching of tree hollows, which disturbs the habitat and such disturbance may result in the death of or damage to any larvae found in the process. In addition, it is impossible to return the habitat to its original condition, thus altering its potential as a suitable environment for *G*. *nobilis* and any other species therein.

Chemical ecological tools such as pheromones can be used as a recording technique for scarab beetles, enabling European countries to detect and, in some cases, map the range of some of these species, thereby proving a valuable technique for monitoring elusive saproxylic beetles [12–18]. Since many species in the Scarabaeidae use pheromones for sexual communication, and as there is an urgent need for a non-destructive detection tool for the conservation of *G*. *nobilis*, we embarked on identification of the potential pheromone of adult beetles in volatile extracts by electrophysiological and behavioural measurements and field trials in the UK. Our research expanded on approaches relating to the preservation of communities of endangered beetle species, such as G. *nobilis*, at sites and landscapes, in particular and relating to the interactions of beetles with their mates and host plants, and was aimed at providing underpinning science for the conservation of *G*. *nobilis* populations, including mitigating the impact of human intervention and climate change. We hypothesised that by considering volatiles produced by both *G*. *nobilis* and its nectaring plants, a more successful monitoring tool would be produced. This is particularly pertinient where the species is rare and numbers are believed to be low.

## Methods and materials

### Preparation of volatile extracts

Two methods were used to sample the headspace of live adult *G*. *nobilis*, after which all beetles were returned to their original habitat: i) Twenty adults (10 females and 10 males) were collected manually from nectaring sites and habitats, over two years from Bystrzyca, Poland (50°55′32.82″N 17°24′35.16″E) and 6 (2 females and 4 males) from Worcestershire, UK (52°06′56.34″N 002°06′46.81″W). Virgin beetles, field-collected before emergence, were sexed using the meta-tibia of the second leg, which is bowed in males, and the pygidium, which is notched in females and kept separately. Sexes were then isolated in a wide-mouthed glass vessel (60 mL) sealed with a rubber septum. The headspace was extracted 3 times for each sample for 20 min with a solid phase micro-extraction (SPME) fibre (Supelco 57342-U), coated with polydimethylsiloxane (PDMS, 100 μm); ii) For dynamic headspace collection (air entrainment), female and male *G*. *nobilis* were placed in separate glass chambers (80 mL, Biochem Glass Apparatus Ltd, UK), with no food or water source, at 20°C/60% RH with a 16:8 hr L:D photoperiod. A filter paper disc (110 mm diam. WhatmanTM, UK), which was put in each chamber to provide a walking substrate for the beetles, was placed on a metal plate of the same diameter and fitted to the chamber with bulldog clips, thereby creating a non-sealed system. For each chamber, air was pumped into the chamber through an activated charcoal filter at 600 mL/min and drawn through a glass tube (8 cm x 0.3 cm i.d.), containing Porapak Q (50 mg) sandwiched between glass wool plugs, at a flow rate of 500 mL/min. In this way, volatile organic compounds (VOCs) were collected continuously for 5 h and eluted from the adsorbent using freshly distilled diethyl ether (750 μL). VOCs were collected from two females and three males. Collected VOC extracts were concentrated to 100 μL under a gentle nitrogen stream and kept at -20°C in tightly capped microvials until use. A VOC extract of the headspace from an empty glass chamber served as negative control to detect system artefacts.

### Electrophysiology

Electroantennogram (EAG) recordings (n = 5) from the antenna of adult male *G*. *nobilis*, without killing the beetles, were made using Ag–AgCl glass electrodes filled with saline solution [19]. The tip of the outermost lamella of an excised antenna was removed to ensure a good contact, and the base of the antenna was inserted into the reference electrode. The outermost lamella was brought into contact with the recording electrode, which was then moved in a way that the lamellae separated, facing the humidified air stream. A short piece of hair was inserted between the middle and innermost lamellae to ensure maximum exposure of olfactory sensilla to the air stream. The signals were passed through a high-impedance amplifier (UN-06; Ockenfels Syntech GmbH, Kirchzarten, Germany). Separation of VOCs collected from adult female *G*. *nobilis* was achieved on a GC (6890N; Agilent Technologies, Santa Clara, CA) equipped with a cool on-column injector and an FID, using a 50 m × 0.32 mm i.d. x 0.52 μm film thickness non-polar HP-1 column. The oven temperature was maintained at 30°C for 2 min and then programmed at 5°C/min to 250°C. The carrier gas was helium. The outputs from the EAG amplifier and the FID were monitored simultaneously and analysed using a customised software package (Syntech). The compound, eventually identified as 2-propyl (*E*)-3-hexenoate, was defined as EAG-active if in five coupled runs it evoked an antennal response distinguishable from background noise. EAG responses (n = 4) were also recorded across a range of doses (0.01, 0.1, 1, 10, 100 and 1000 μg) of 2-propyl (*E*)-3-hexenoate after identification, loaded onto small pieces of filter paper. These were then placed inside Pasteur pipettes, and applied at 30 min intervals into the humidified air stream flowing towards the antenna, using a stimulus controller. Antennal responses were normalized against a solvent (hexane), which was delivered before and after the set of stimuli.

### Chemical analysis of extracts

VOCs collected by SPME Fibres were analysed by coupled gas chromatography-mass spectrometry (GC-MS), using a 5890 Series II Plus GC, fitted with an Agilent DB5-MS column (25 m x 0.25 mm i.d. x 0.25 μm film thickness), coupled to an Agilent 5970 MSD. Each fibre was desorbed for 2 min in splitless mode, inlet temperature 240°C and transfer line temperature 280°C. The oven temperature was initially 50°C (2 min), then programmed to rise at 10°C/min to 280°C (5 min). Collected GC data were examined using AMDIS 32 deconvolution software and NIST 14 mass spectra library (www.nist.gov). VOC extracts were analysed on a HP 6890 GC, equipped with a cool-on-column injector and flame ionization detector (FID), and fitted with a 50 m x 0.32 mm i.d. x 0.52 μm film thickness non-polar HP-1 column (J & W Scientific), as well as a 30 m x 0.32 mm i.d. x 0.5 μm film thickness polar DB-WAX column (J & W Scientific). The oven temperature was maintained at 30°C for 1 min, then programmed to rise at 5°C/min to 150°C and held for 0.1 min, then programmed to rise at 10°C/min to 250°C and held for 20 min. The carrier gas was hydrogen. Quantification of compounds was achieved using the single-point external standard method with a series of C7-C22 alkanes. For tentative identification of the EAG-active peak from female air entrainment extracts, an Agilent 6890N GC fitted with a capillary GC column (50 m × 0.32 mm i.d, 0.52 μm film thickness non-polar HP-1 column), and equipped with a cool on-column injector, was directly coupled to a magnetic sector mass spectrometer (Micromass Autospec Ultima; Waters, Milford, MA). Ionization was by electron impact at 70 eV, 220°C. The GC oven temperature was maintained at 30°C for 1 min, then programmed to rise at 5°C/min to 250°C, total run time 70 min. Tentative identifications were made by comparison of mass spectra with library database [20], and confirmed by peak enhancement using an authentic standard on non-polar and polar GC columns.

One of the VOC extracts obtained from females was identified by GC analysis to be suitable for NMR, as the target peak comprised 90% of the total solute. The solvent was completely evaporated under a gentle stream of nitrogen, and the residue dissolved in deuterated dichloromethane (CD_2_Cl_2_, 15 μL). The sample was transferred and sealed in a capillary NMR tube. ^1^H NMR data were collected with a Bruker Avance 500 MHz NMR spectrometer equipped with a 1 mm TXI capillary probe in CD_2_Cl_2_, referenced to 5.35 ppm.

### Chemical synthesis of 2-propyl (*E*)-3-hexenoate

Since 2-propyl (*E*)-3-hexenoate is a novel compound that is not commercially available, it was synthesised to ensure enough material was available for structure conformation, bioassays and field trials. (*E*)-3-Hexenoic acid (5.32 g, 46.5 mmol), 2-propanol (14 g, 233 mmol) and conc. sulphuric acid (0.46 cm^3^) were heated under reflux for 2 h. The reaction mixture was poured into ice water (100 cm^3^) and extracted with petroleum ether bp 40–60°C. The extract was washed with water, sodium bicarbonate solution twice, and water, and concentrated by rotary evaporation. The residue (4.4 g) was distilled at 60°C under water pump vacuum to yield a colourless liquid (3.9 g, 54%). The product gave a single peak on GC with KI 1026 (HP-1), 1293 (DB-WAX) and 1063 (DP-5). IR v_max_/cm: 2979 and 2935 (C-H), 1374 (C-H), 1732 (C = O), 1169 and 1108 (C-O); ^1^H NMR δ_H_ ppm (CDCl_3_, 500 MHz) 5.60 (1H, dt, *J* = 15.4, 6.1 Hz, H-4), 5.51 (1H, dt, *J* = 15.4, 6.7 Hz, H-3), 5.00 (1H, septet, *J* = 6.3 Hz, H-2 propyl), 2.98 (2H, dd, *J* = 6.7, 0.9 Hz, H-2), 2.04 (2H, m, H-5), 1.24 (6H, d, *J* = 6.3 Hz, H-1 propyl), 0.99 (3H, t, *J* = 7.5 Hz, H-6); ^13^C NMR δ_C_ ppm (125 MHz, CDCl_3_): 171.86 (C-1), 136.13 (C-4), 120.8 (C-3), 67.77 (C-2 propyl), 38.43 (C-2), 25.53 (C-5), 21.82 (C-1 propyl), 13.51 (C-6); EIMS: m/z 156 (M^+^, 2.5%), 114 (27), 97 (7.9), 69 (44), 43 (100), 41 (51), 39 (20).

### Y-tube olfactometry

Y-tube olfactometry experiments were carried out using individual adult *G*. *nobilis*. The following experiments were conducted: (i) live beetles vs control air; (ii) the headspace of *H*. *sphondylium* flower heads vs control air; (iii) synthetic 2-propyl (*E*)-3-hexenoate vs solvent control; (iv) headspace of flower heads + synthetic 2-propyl (*E*)-3-hexenoate vs solvent control. When offering live beetles as the stimulus, a single individual was placed in one arm of the olfactometer. One flower head/replicate was used. 2-Propyl (*E*)-3-hexenoate was tested at doses of 1, 10 and 100 ng, and 1, 10 and 100 μg. When tested together, 1 flower head and 100 μg of synthetic 2-propyl (*E*)-3-hexenoate was placed in one arm of the olfactometer. For each replicate, an individual beetle was released at the base of the Y-tube, observed for 5 min and the choice made was recorded. The air flow within the olfactometer was 400 mL/min. Throughout all experiments, no beetle was used more than once and the olfactometer was cleaned thoroughly between trials to avoid contamination. Olfactometry data were analysed in R 3.4.3 [21], using the binomial exact test.

### Live field trapping

Field trials were set up near Pershore, UK (52°06′56.34″N 02°06′46.81″W), in an ca. 80-year-old plum orchard, (permission granted by Worcestershire Wildlife Trust)and the New Forest, (permission granted by Natural England) a mixed broadleaf forest and woodland pasture (50°52′21.20″N 01°34′36.80″W). Synthetic 2-propyl (*E*)-3-hexenoate (150 ul) was formulated into capped, pointed polyethylene vials (0.2 mL PCR tube, Starlab 11402–8100) that had a 1 mm diam. pre-drilled hole in the lid. The mean release rate 0.29 mg/h) was calculated by weight-loss measurements at 20°C from five dispensers over a 24 h period. Bait dispensers were fixed onto the transparent upper panel of CSALOMON VARb3 funnel traps (produced by the Plant Protection Institute, CAR HAS, Budapest, Hungary; www.csalomontraps.com), which were hung up on branches at ca. 2 m height. In five replications at each site, the following treatments were tested: i) 2-propyl (*E*)-3-hexenoate; ii) 1 flower head of *H*. *sphondylium*); iii) combination of i) and ii); iv) control traps with empty vials. The lures were topped up weekly. Trials were run from 30th May to 30th June 2017 in Pershore, and from 20^th^ June to 5^th^ July 2018 in the New Forest. The latter was a volunteer-run trial and only conditions i), iii) and iv) were sited. Traps were inspected every other day, when caught *G*. *nobilis* beetles were recorded, marked and released. The data units for the field experiments were number of insects caught/trap. As raw data did not fulfil the requirements for parametric statistics, capture data were analysed by a Kruskal-Wallis one-way analysis of variance, followed by pairwise comparisons by Dunn’s test. All statistical procedures were conducted using R 3.4.3 and the Dunn test package (Dinno 2017).

## Results

**Fig 1:**
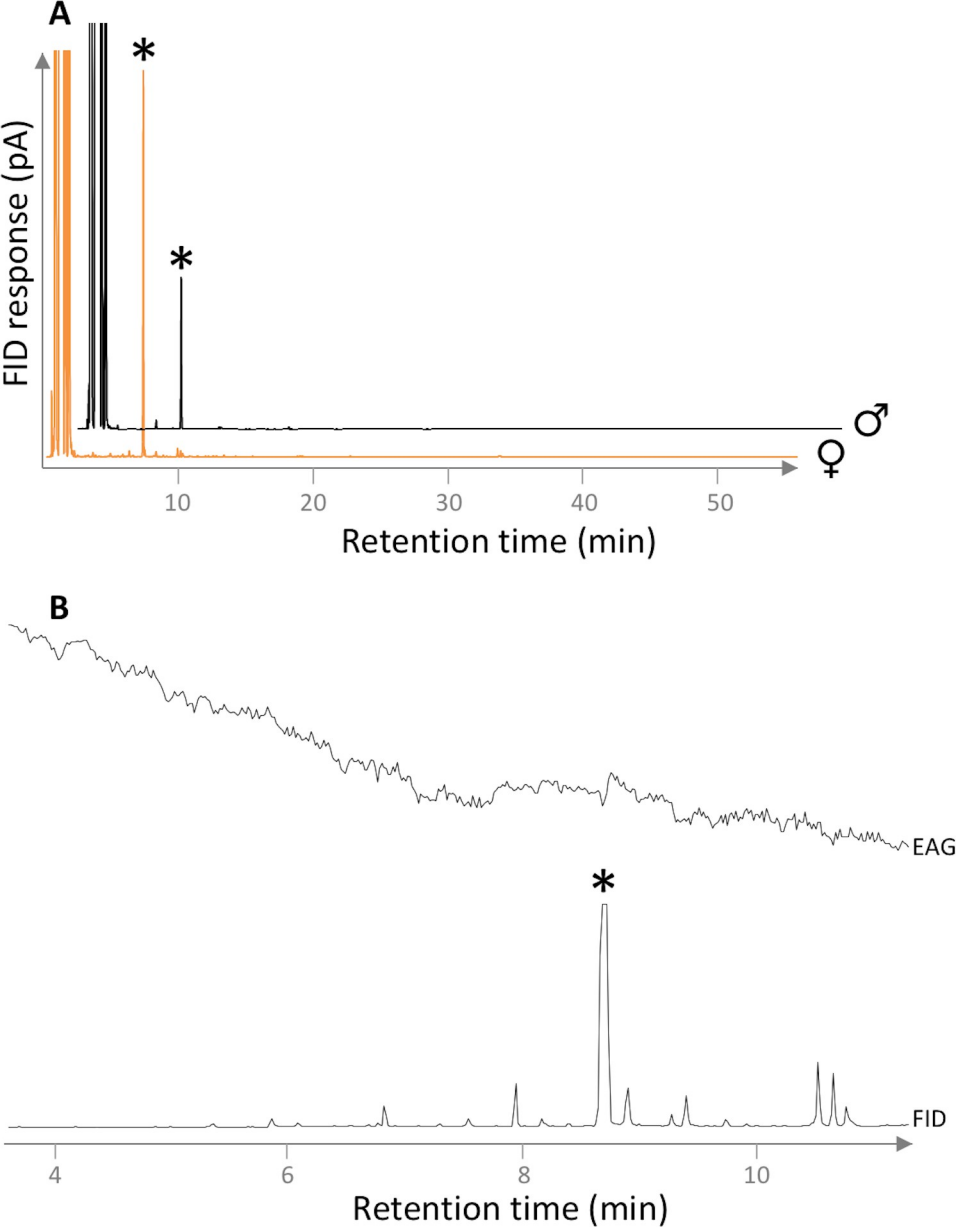
**A** Gas chromatography (GC) analysis of volatile organic compound (VOC) extracts collected by air entrainment from noble chafers, *Gnorimus nobilis*, on a non-polar HP-1 column (traces shifted along both axes, but on same scale). **B** coupled GC-EAG responses of male *G*. *nobilis* to a region of a female VOC extract on a non-polar HP-1 GC column. Asterisk marks peak with reproducible EAG activity. Vertical line indicates 0.2 mV for the EAG output.

**Fig 2:**
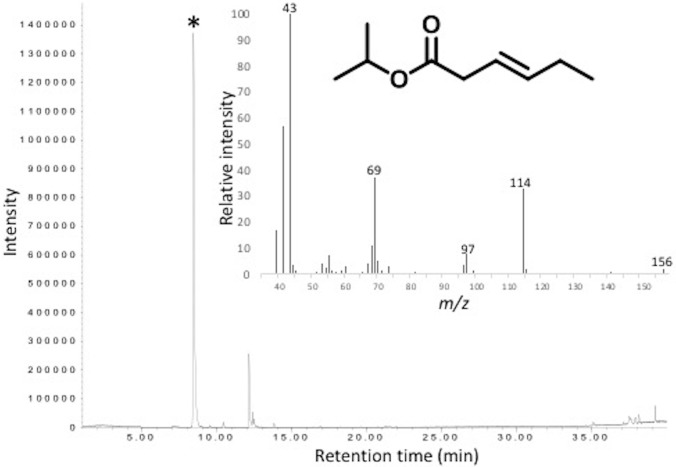
Coupled GC-mass spectrometry (GC-MS) analysis of a VOC extract collected from female *Gnorimus nobilis* by solid-phase microextraction (SPME). Insert: 70eV mass spectrum of unknown compound identified as 2-propyl (*E*)-3-hexenoate (marked with asterisk on TIC).

Gas chromatographic (GC) analysis of VOCs collected from virgin female and male *G*. *nobilis* by air entrainment revealed the presence of a major component in high purity with a GC retention unit value of 1026 (50 m HP-1 column) (Fig 1A), which elicited electrophysiological activity from male antennae (Fig 1B, S1 Fig). Coupled GC-mass spectrometry (GC-MS) analysis of a female VOC extract collected by SPME revealed the presence of the same component (Fig 2), the structure of which was unknown. The 70eV EI mass spectrum (Fig 2) showed a base peak at *m/z* 43 and a molecular ion at *m/z* 156. Diagnostic fragments at *m/z* 114 (M+ -42, i.e., McLafferty rearrangement), *m/z* 97 (M+ -59, loss of a propoxy group) and *m/z* 69 (M+ -87, loss of a propoxycarbonyl group) indicated the compound to be a propyl ester of a C_6_-carboxylic acid with one double bond equivalent. Capillary-probe ^1^H NMR analysis of a female VOC extract collected by air entrainment confirmed the presence of double bond resonances and signals commensurate with those expected adjacent to a double bond or carbonyl moiety (2–3 ppm). The large solvent peaks and other impurities made identifying which signals were relevant to the GC peak impossible. A COSY spectrum revealed the potential presence of a pair of spin systems, and these were investigated by 1-dimensional TOCSY experiments, showing a spin system that was consistent with a hex-3-enoyl moiety, and a second with a 2-propyl system (S2 Fig). Examination of the olefinic coupling constants revealed a large coupling constant of 15.4 Hz between the two olefin protons, indicative of a *trans* alkene geometry (S3 and S4 Figs). Tentative identification of the unknown compound was confirmed as 2-propyl (*E*)-3-hexenoate by comparison of NMR spectra, GC and MS data with an authentic sample prepared by chemical synthesis. Using the authentic standard, female and male beetles were estimated to produce ca. 3.5 μg/individual/hr and 2.5 μg/individual/hr of 2-propyl (*E*)-3-hexenoate, respectively.

**Fig 3:**
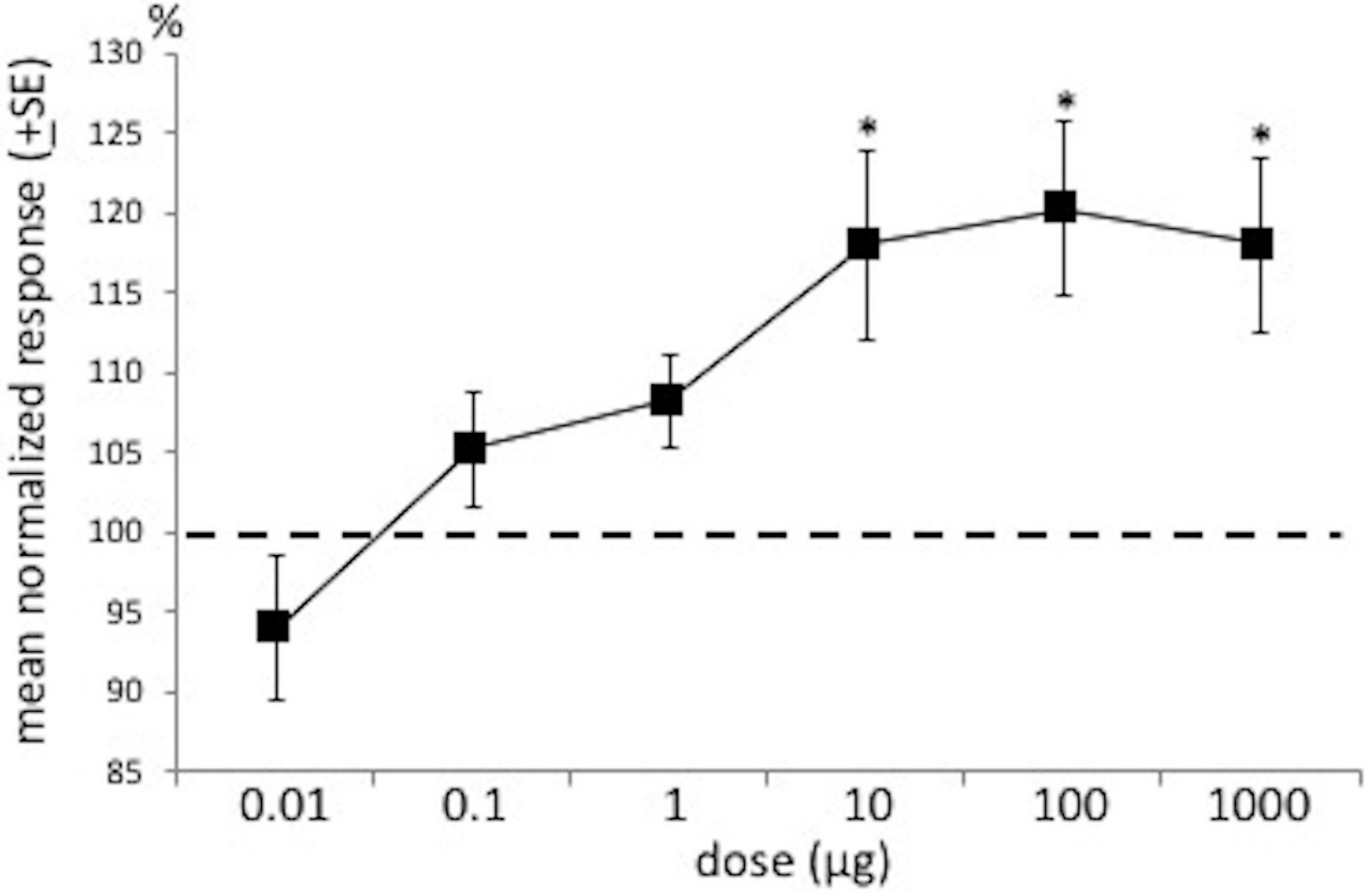
EAG responses of male *Gnorimus nobilis* antennae to different doses of synthetic 2-propyl (*E*)-3-hexenoate. Dashed line indicates level of antennal responses to the hexane control (100%), against which all responses were normalized. Asterisks mark doses evoking antennal responses significantly different from the control (ANOVA, followed by LSD test at 5% level; 4 replicates).

Y-tube olfactometer bioassays, using adult male and female *G*. *nobilis* beetles, showed that males preferred the arm containing the odour of either a female or male in separate experiments (Table 1, S1 Table). Female *G*. *nobilis* beetles, however, were repelled by the arm containing female or male odour (Table 1). Synthetic 2-propyl (*E*)-3-hexenoate elicited EAG responses from male antennae in a dose-dependent manner (Fig 3, S2 Table). In Y-tube olfactometer assays, males responded to the synthetic compound at doses of 1 μg and above (Table 1), whereas females were repelled by the compound at all doses tested. Both sexes preferred the odour from *H*. *sphondylium* flowers compared to the control arm (Table 1, S1 Table). Addition of synthetic 2-propyl (*E*)-3-hexenoate to the odour of flowers resulted in male responses to the combination being enhanced compared to either flower or compound when tested alone, whereas addition of the compound to the odour of flowers resulted in repellence in females (Table 1, S1 Table).

**Table 1:** Olfactory responses of adult noble chafers, *Gnorimus nobilis*, to conspecific odours, different doses of 2-propyl (*E*)-3-hexenoate (2P3H), and to combinations of 2P3H (100 μg) and host (floral) material in a Y-tube olfactometer.

	Males			Females		
Stimulus	Pos^*a*^	No. replicates^*b*^	*p*	Pos	No. replicates	*p*
**Opposite sex**	8	8	0.0001	0	5	0.0001
**Same sex**	8	8	0.0001	0	5	0.0001
**2P3H (1 ng)**	2	17	0.0012	0	12	0.0001
**2P3H (10 ng)**	1	17	0.0001	1	12	0.0032
**2P3H (100 ng)**	1	17	0.0003	0	12	0.0001
**2P3H (1 μg)**	12	17	0.0717	0	12	0.0001
**2P3H (10 μg)**	13	17	0.0245	2	12	0.0193
**2P3H (100 μg)**	17	17	0.0001	0	12	0.0001
***H*. *sphondylium***	15	17	0.0012	10	12	0.0001
**2P3H + *H*. *sphondylium* vs *H*. *sphondylium* (Co)**	14	17	0.0064	0	12	0.0001
**2P3H + *H*. *sphondylium* vs 2P3H (Co)**	11	17	0.1662	2	12	0.0001

^*a*^ Pos is number of beetles showing positive response, measured as the choice of olfactometer arm by an individual insect at the end of each replicate experiment.

^*b*^ No. replicates is the total number of beetles tested individually to each stimulus. Full raw data, including a breakdown of non-responders and beetles which went to the control arm of the olfactometer, is available in Supplementary Materials.

Data were analysed by the exact binomial test against chance level (p = 0.5). This means that where p < 0.05 and *Pos* is more than half of the total number of replicates, beetles showed a significant attraction to the tested stimulus (e.g., for male beetles to the opposite sex, first line); where *p* < 0.05 and *Pos* is less than half of the total number of replicates, beetles showed no behavioural response to the tested stimulus (e.g., for female beetles to the opposite sex, first line).

**Fig 4:**
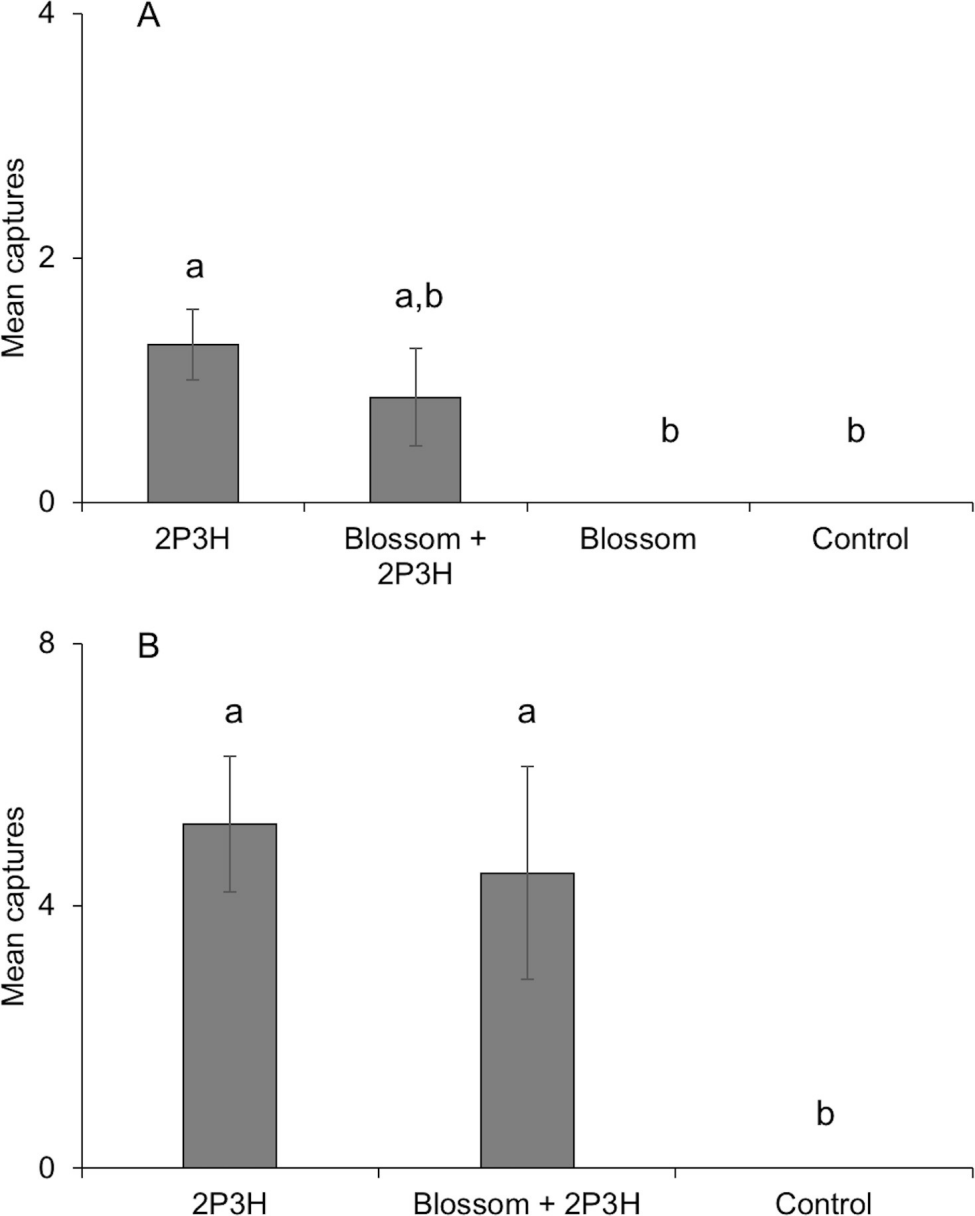
Mean capture per trap on each trapping occasion of male *Gnorimus nobilis* in field trials at A Pershore, UK and B New Forest, UK. Traps were baited with 150 mg synthetic 2-propyl (*E*)-3-hexenoate (2P3H), 1 flowerhead of *Heracleum sphondylium*, and their combination. Unbaited traps were used as control. Columns with same letter are not significantly different (Kruskal-Wallis, Dunn`s test). Total catches: Worcester = 18, New Forest = 78.

In live field trapping trials in the UK, a total of 96 separate male *G*. *nobilis* were caught in traps; 18 in Pershore and 78 in the New Forest. No beetles were observed outside of the traps at any of the sites despite searching. For both sites, captures were significantly different across lure types (Worcester χ^2^(3) = 14.07, *P* < 0.01, Fig 4A; New Forest χ^2^(2) = 22.52, *P* < 0.01, Fig 4B, S3 Table). In Pershore, traps baited with 2-propyl (*E*)-3-hexenoate caught significantly more specimens than either the blossom alone (z = -3.14, *P <* 0.001) or control traps (z = -3.14, P < 0.01). There were no significant differences between any of the other treatments (all comparisons *P* > 0.1). In the New Forest, traps baited with 2-propyl (*E*)-3-hexenoate alone and with its binary combination with *H*. *sphondylium* attracted significantly more beetles than control traps (lure only z = -4.32, *P* < 0.001, lure and blossom z = 3.30, *P* = 0.002).

## Discussion

We used chemically synthesized 2-propyl (*E*)-3-hexenoate to create a platform for the development of a monitoring scheme for *G*. *nobilis*. To date, detection of this extremely rare beetle, with conservation status across its European range, has largely relied on detection of larval frass in tree hollows, or chance sightings of the adult. The former method may result in disturbance of habitats and, since the frass is slow to decay, possible inaccuracies in presence data. Sightings of the adult are uneven and more unreliable as a method of monitoring, and it should be noted that during all trials conducted here, searching for adults did not elicit any specimens, which alone would have led to the erroneous suggestion that the beetle was no longer present. Our results are particularly pertinent for the New Forest, where there were only 20 confirmed records since 1894 [22]. This is why our replicates for olfactometry were so low but, more importantly, the fact that 78 beetles were caught in 15 days shows the efficacy of the compound and demonstrates the validity of its use in future field surveys.

Volatile pheromones play a critical role in insect biology and have long been explored as tools for management of pest species. By comparison, they have been less utilized for insects of conservation status, only emerging over the last 15 years as a new approach that applies research outputs to the conservation of insect biodiversity [23], including mitigating the impact of human intervention and climate change. Pheromones have been isolated and used successfully as a monitoring tool for *Osmoderma eremita* Scopoli (Coleoptera: Scarabaeidae) [15], *Elater ferrugineus* L. (Coleoptera: Elateridae) [12,17], *Rosalia alpina* L. (Coleoptera: Cerambycidae) [18] and *Graellsia isabellae* Graëlls (Leptidoptera: Saturniidae) [24], enabling European countries to detect and, in some cases, map the range of some of these species, thereby proving a valuable technique for monitoring elusive saproxylic beetles [13,15]. Since several members of the Scarabaeidae use volatile pheromones for sexual communication [25], and due to the urgent need for a non-destructive detection tool for the conservation of the noble chafer, *G*. *nobilis*, a saproxylic species that is vulnerable across its Europe-wide distribution, we embarked on a study of the behavioural and chemical ecology of this species. Using a combination of behaviour, electrophysiology, analytical and field experiments, we showed that *G*. *nobilis* utilizes a volatile compound, 2-propyl (*E*)-3-hexenoate, as an essential part of its life-cycle, and propose that this compound is the only pheromone component, on the basis of GC-EAG results. Although the pheromone is produced by both sexes in large amounts, only males demonstrate a positive behavioural response. This peculiar pattern of chemical signalling is the opposite to the biological activity of the aggregation pheromone produced by the Cerambycid beetle *Rosalia alpina*, since in that case, the compound was only produced by males and attracted both sexes [18]. The production of 2-propyl (*E*)-3-hexenoate is predicted to be metabolically costly for *G*. *nobilis*, whose only food source as an adult is believed to be nectar [26]. Such a response to a volatile pheromone may reflect an intermediate evolutionary stage towards developing into a sex-specific signal [27]. Similar, although not identical, evolving sexual communication systems have been found in *Holotrichia consaguinea* Blanchard (Coleoptera: Scarabaeidae) [28] and in *Agriotes sordidus* Illiger (Coleoptera: Elateridae) [29]; however, in these cases, both females and males were attracted to the female-produced pheromone. Leal et al [28] proposed that for *H*. *consaguinea*, the pheromone was yet to become sex-specific. According to Leal [30], in some species of the Melolonthinae, including *H*. *consanguinea* and *H*. *parallela* Motschulsky, sex pheromones evolved from a primarily defensive role, evidenced by the large released amounts and their antimicrobial activity. This was also shown in a wasp, emphasizing the parsimonious use of the same semiochemical for intra- and intersexual communication and defence [31]. Demonstration of an additional defensive function for 2-propyl (*E*)-3-hexenoate in *G*. *nobilis* may support such hypotheses on the direction of signal evolution. Ester compounds are described as pheromone components in phylogenetically distant taxa of the Scarabaeidae. Female-produced, amino acid-derived methyl esters are widespread in the Melolonthinae subfamily, whereas ethyl 4-methyloctanoate is a male-produced aggregation pheromone constituent of a number of species in the Dynastinae subfamily [25]. The discovery that *G*. *nobilis* also utilizes an ester for intraspecific sexual communication indicates that this class of compounds have gained similar functions during the evolution of different lineages of the Scarabaeidae.

The classification of the compound, based on the current semiochemical terminology [32], is somewhat problematic, but in our view, it should be termed an aggregation pheromone [33] as a consequence of the production pattern. The fact that 2-propyl (*E*)-3-hexenoate is emitted in large amounts also indicates that it may act together with habitat volatiles to signal the presence of mating sites to conspecific males [34]. However, it is an open question why there is such a remarkable difference between female and male responses to the pheromone and plant volatiles. In laboratory bioassays, females were repelled by the pheromone, and when it was added to otherwise attractive plant odours, it curtailed positive responses. It is possible that it aids the dispersal of egg-laying females in the habitat, seeking hollow trees which are in short supply, to avoid the negative effects of high-density breeding sites on larval survival. Thus, a possible volatile-mediated mate-finding scenario for *G*. *nobilis* is that freshly emerged beetles utilize plant volatiles, among other floral cues, to find feeding sites. 2-Propyl (*E*)-3-hexenoate may signal to mated females the presence of a high number of individuals at a particular location, and force them to disperse to neighbouring feeding sites. Males, however, are attracted to feeding females and males and the pheromone helps them distinguish conspecifics from similar Cetoniinae species, e.g., *Cetonia a*. *aurata* L. or *Potosia cuprea* Scop. Mate recognition might occur via contact semiochemicals [35]. Although not investigated in this study, female *G*. *nobilis* likely possess the sensory capabilities to detect the pheromone (autodetection, [36]), as it was recently described for other members of the Coleoptera [29].

In terms of lure development, future studies may focus on the identification of floral compounds from nectar plants of *G*. *nobilis*, e.g., *S*. *nigra*, that may elicit synergism when applied together with the pheromone. A combined synthetic lure would make the frequent replacement of live plant material in the traps unnecessary and would extend the longevity of the attractant in the monitoring device. However, the necessity for field observation as a pre-requisite for successful lure development must be emphasized, especially where the ecological characteristics of the species vary across its distribution. Where possible, the development of lures should be a two-pronged procedure involving both the collection of volatiles and field-based behavioural studies. Another possible direction for the improvement of trap performance is research into the colour sensitivity of *G*. *nobilis*. Several diurnal members of the Cetoniinae respond to colour [37], but not to the circularly polarizing exocuticle of conspecifics [38]. In fact, the appropriate trap colour can greatly enhance activity of the volatile lure in several scarab species [37,39], which might also be the case in *G*. *nobilis*.

In summary, our study has provided underpinning science for the conservation of endangered *G*. *nobilis* populations at sites in the UK, including mitigating the impact of human intervention and climate change. The study forms the basis for non-invasive surveillance of the cryptic *G*. *nobilis*, potentially enabling detection of live specimens without the need for habitat searching or disturbance, and continuous human monitoring. Furthermore, as the synthetic pheromone is relatively simple to produce and can be applied in an already existing trap design, the platform is set for the development of a low-cost detection and monitoring tool. An obvious drawback of a live trapping device is the need constantly to remove captured specimens from the traps. However, as the detection of the species only requires a short period during its swarming, it might not prove to be overly labour-intensive. Further efforts will focus on the identification of plant-based attractants, as well as the effect of colour on beetle behaviour, and will test combinations of olfactory and visual cues for maximum trapping efficacy.

## Ethics

Permission to monitor the species within UK orchards was obtained from the Worcestershire Wildlife Trust. The permissions to monitor in the New Forest was granted by Natural England.

## Data accessibility

Supplementary material is available on NMR analyses, as well as EAG, GC-EAG, olfactometry and field data. GC data is available on Figshare: doi 10.6084/m9.figshare.7223453.v1

## Supporting information

S1 Fig Coupled GC-EAG traces of five male Gnorimus nobilis antennae from five individuals. The most abundant peak is 2-propyl (E)-3 hexenoate.
(TIFF)

S2 Fig NMR analyses of natural and synthetic 2-propyl (E)-3-hexenoate. (TIFF)

S3 Fig Geometry of trans alkene. ^1^H NMR of natural 2-propyl (*E*)-3-hexenoate: δ_H_ (CD_2_Cl_2_, 500 MHz) 5.60 (1H, dt, *J* = 15.4, 6.1 Hz, H-4), 5.51 (1H, dt, *J* = 15.4, 6.7 Hz, H-3), 5.00 (1H, septet, *J* = 6.3 Hz, H-2 propyl), 2.98 (2H, dd, *J* = 6.7, 0.9 Hz, H-2), 2.04 (2H, m, H-5), 1.24 (6H, d, *J* = 6.3 Hz, H-1 propyl), 0.99 (3H, t, *J* = 7.5 Hz, H-6); m/z (EI) 156 (M^+^, 1), 114 (8), 97 (2), 81 (1), 69 (24), 55 (5), 53 (7), 43 (100), 41 (56).
(TIFF)

S4 Fig Coupling constants of the olefinic region of the ^1^H-NMR. (TIFF)

S1 Table Olfactometry data. (DOCX)

S2 Table EAG data. (DOCX)

S3 Table Field trapping data. (DOCX)

## References

[1] Lachat T, Müller J. Importance of Primary Forests for the Conservation of Saproxylic Insects. Springer, Cham; 2018 pp. 581–605. 10.1007/978-3-319-75937-1_17

[2] EEA. EU 2010 biodiversity baseline, Tehcnical Report no. 12/2020. 2010.

[3] Speight M. Saproxylic invertebrates and their conservation. Nature and Environment Series, No. 42. Strasbourg; 1989.

[4] Alexander K. The special importance of traditional orchards for invertebrate conservation, with a case study of the BAP priority species the Noble Chafer Gnorimus nobilis In: Rotherham ID, editor. Orchards and Groves: Their History, Ecology, Culture and Archaeology, Landscape Archaeology and Ecology. Sheffield: Wildtrack Publishing; 2008 pp. 11–17.

[5] Nieto A, Alexander K. European Red List of Saproxylic Beetles IUCN Species Programme. Luxembourg: Publications Office of the European Union; 2010 10.2779/84561

[6] Campanaro A, Zapponi L, Hardersen S, Méndez M, Al Fulaij N, Audisio P, et al A European monitoring protocol for the stag beetle, a saproxylic flagship species. Leather SR, Müller J, editors. Insect Conserv Divers. 2016;9: 574–584. 10.1111/icad.12194

[7] Harvey D, Gange A, Hawes C, Rink M. Bionomics and distribution of the stag beetle, Lucanus cervus, across Europe. Insect Conserv Divers. 2011;4: 23–38.

[8] BRIG. Report on the Species and Habitat Review to the UK Standing Committee. 2007.

[9] Alexander K. Revision of the Index of Ecological Continuity as used for saproxylic beetles. English Nat Res Reports. 2004;467.

[10] Lamellicornia Endrödi S. Fauna Hungariae IX/4. Budapest: Akadémiai Press; 1956.

[11] Alexander K. The invertebrates of living & decaying timber in Britain and Ireland—a provisional annotated checklist. English Nature Research Reports 467. Peterborough; 2002.

[12] Harvey D, Harvey H, Harvey R, Kadej M, Hedenström E, Gange A, et al Use of novel attraction compounds increases monitoring success of a rare beetle, Elater ferrugineus. Insect Conserv Divers. 2017;10: 161–170. 10.1111/icad.12214

[13] Harvey DJ, Harvey H, Larsson MC, Svensson GP, Hedenström E, Finch P, et al Making the invisible visible: determining an accurate national distribution of Elater ferrugineus in the United Kingdom using pheromones. Didham R, Müller J, editors. Insect Conserv Divers. 2017;10: 283–293. 10.1111/icad.12227

[14] Kadej M, Zając K, Ruta R, Gutowski JM, Tarnawski D, Smolis A, et al Sex pheromones as a tool to overcome the Wallacean shortfall in conservation biology: a case of Elater ferrugineus Linnaeus, 1758. J Insect Conserv. 2014;19: 25–32. 10.1007/s10841-014-9735-4

[15] Larsson M, Hedin J, Svensson G, Tolasch T, Francke W. Characteristic odor of Osmoderma eremita identified as a male-released pheromone. J Chem Ecol. 2003;29: 575–587. 10.1023/A:1022850704500 12757320

[16] Larsson M, Svensson G. Pheromone monitoring of rare and threatened insects: exploiting a pheromone-kairomone system to estimate prey and predator abundance. Conserv Biol. 2009;23: 1516–25. 10.1111/j.1523-1739.2009.01263.x 19508672

[17] Svensson G, Larsson M, Hedin J. Attraction of the larval predator Elater ferrugineus to the sex pheromone of its prey, Osmoderma eremita, and its implication for conservation biology. J Chem Ecol. 2004;30: 353–363. 10.1023/B:JOEC.0000017982.51642.8c 15112729

[18] Žunič Kosi A, Zou Y, Hoskovec M, Vrezec A, Stritih N, Millar JG. Novel, male-produced aggregation pheromone of the cerambycid beetle Rosalia alpina, a priority species of European conservation concern. PLoS One. 2017;12: 1–19. 10.1371/journal.pone.0183279 28827817 PMC5565183

[19] Maddrell S. Secretion by the Malpighian tubules of Rhodnius. The movements of ions and water. J Exp Biol. 1969;51: 71–97.

[20] NIST. NIST/EPA/NIH Mass Spectral Library. 2008.

[21] R Core Team. R: A language and environment for statistical computing. Vienna; 2017.

[22] NBN Gateway. NBN Gateway Gnorimus nobilis records [Internet]. 2017 [cited 30 Mar 2015]. Available: https://data.nbn.org.uk/Taxa/NBNSYS0000010968

[23] Leal WS. Reverse chemical ecology at the service of conservation biology. Proc Natl Acad Sci. 2017;114: 12094–12096. 10.1073/pnas.1717375114 29093161 PMC5699103

[24] Millar JG, McElfresh JS, Romero C, Vila M, Marí-Mena N, Lopez-Vaamonde C. Identification of the Sex Pheromone of a Protected Species, the Spanish Moon Moth Graellsia isabellae. J Chem Ecol. 2010;36: 923–932. 10.1007/s10886-010-9831-1 20658260 PMC2941043

[25] Vuts J, Imrei Z, Birkett M, Pickett JA, Woodcock CM, Tóth M. Semiochemistry of the Scarabaeoidea. J Chem Ecol. 2014;40: 190–210. 10.1007/s10886-014-0377-5 24474404

[26] Johansson BG, Jones TM. The role of chemical communication in mate choice. Biol Rev. Wiley/Blackwell (10.1111); 2007;82: 265–289. 10.1111/j.1469-185X.2007.00009.x 17437561

[27] Wyatt TD. Pheromones and signature mixtures: defining species-wide signals and variable cues for identity in both invertebrates and vertebrates. J Comp Physiol A. Springer-Verlag; 2010;196: 685–700. 10.1007/s00359-010-0564-y 20680632

[28] Leal W, Yadava C, Vijayvergia J. Aggregation of the scarab beetle Holotrichia consanguinea in response to female-released pheromone suggests secondary function hypothesis for semiochemical. J Chem Ecol. 1996;22: 1557–1566. 10.1007/BF02027731 24226255

[29] Tóth M, Furlan L, Vuts J, Szarukán I, Ujváry I, Yatsynin V, et al Geranyl hexanoate, the female-produced pheromone of Agriotes sordidus Illiger (Coleoptera: Elateridae) and its activity on both sexes. Chemoecology. 2015;25: 1–10. 10.1007/s00049-014-0170-5

[30] Leal WS. Evolution of Sex Pheromone Communication in Plant-Feeding Scarab Beetles In: Cardé R, Minks A, editors. Insect Pheromone Research. Boston, MA: Springer US; 1997 pp. 505–513. 10.1007/978-1-4615-6371-6_43

[31] Weiss I, Rössler T, Hofferberth J, Brummer M, Ruther J, Stökl J. A nonspecific defensive compound evolves into a competition avoidance cue and a female sex pheromone. Nat Commun. 2013;4: 1767 10.1038/ncomms2748 24231727 PMC3868268

[32] Cardé RT. Defining Attraction and Aggregation Pheromones: Teleological Versus Functional Perspectives. J Chem Ecol. 2014;40: 519–520. 10.1007/s10886-014-0465-6 24946748

[33] Wertheim B, van Baalen E-JA, Dicke M, Vet LEM. Pheromone-mediated aggregation in nonsocial arthopods: An evolutionary ecological perspective. Annu Rev Entomol. 2005;50: 321–346. 10.1146/annurev.ento.49.061802.123329 15355243

[34] Xu H, Turlings TCJ. Plant Volatiles as Mate-Finding Cues for Insects. Trends Plant Sci. 2018;23: 100–111. 10.1016/j.tplants.2017.11.004 29229187

[35] Fombong A, Teal P, Arbogast R, Ndegwa P, Irungu L, Torto B. Chemical Communication in the Honey Bee Scarab Pest Oplostomus haroldi: Role of (Z)-9-Pentacosene. J Chem Ecol. 2012;38: 1463–1473. 10.1007/s10886-012-0211-x 23149473

[36] Holdcraft R, Rodriguez-Saona C, Stelinski L. Pheromone Autodetection: Evidence and Implications. Insects. Multidisciplinary Digital Publishing Institute; 2016;7: 17 10.3390/insects7020017 27120623 PMC4931429

[37] Vuts J, Kaydan MB, Yarimbatman A, Tóth M. Field catches of Oxythyrea cinctella using visual and olfactory cues. Physiol Entomol. Wiley/Blackwell (10.1111); 2012;37: 92–96. 10.1111/j.1365-3032.2011.00820.x

[38] Blahó M, Egri Á, Hegedüs R, Jósvai J, Tóth M, Kertész K, et al No evidence for behavioral responses to circularly polarized light in four scarab beetle species with circularly polarizing exocuticle. Physiol Behav. 2012;105: 1067–1075. 10.1016/j.physbeh.2011.11.020 22155007

[39] Schmera D, Tóth M, Subchev M, Sredkov I, Szarukán I, Jermy T, et al Importance of visual and chemical cues in the development of an attractant trap for Epicometis (Tropinota) hirta Poda (Coleoptera: Scarabaeidae). Crop Prot. 2004;23: 939–944. 10.1016/j.cropro.2004.02.006

