# Supplementary figures and images for: Environmentally vulnerable noble chafers exhibit unusual pheromone-mediated behaviour
Source: PLoS One. 2018 Nov 1;13(11):e0206526. doi: 10.1371/journal.pone.0206526 (PMC6211686; doi:10.1371/journal.pone.0206526)

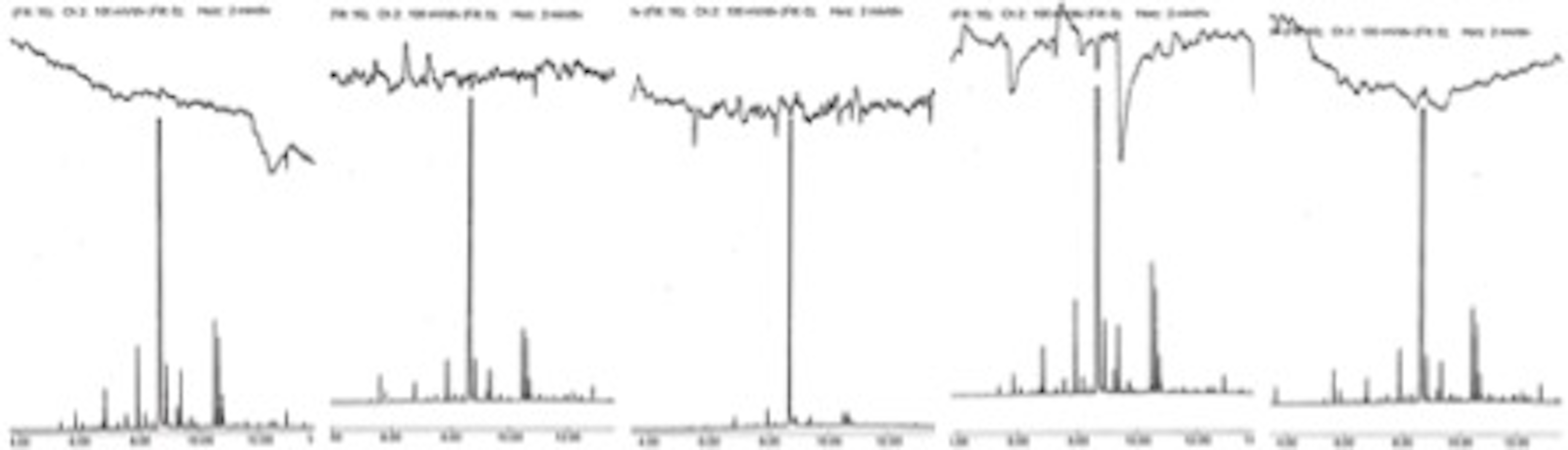

Supplement: S1 Fig — The most abundant peak is 2-propyl (E)-3 hexenoate. (TIFF) [file pone.0206526.s001.tiff]

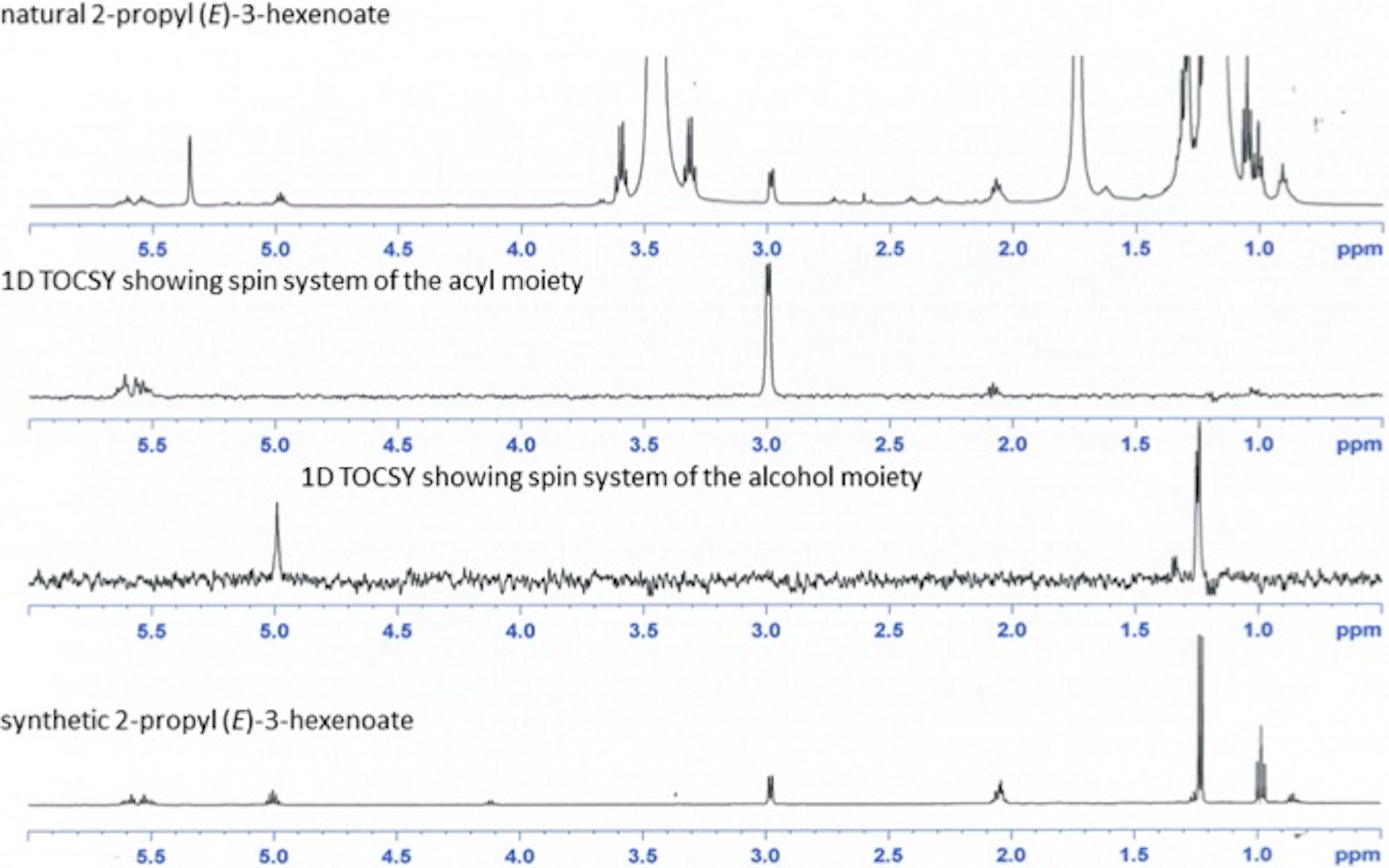

Supplement: S2 Fig — (TIFF) [file pone.0206526.s002.tiff]

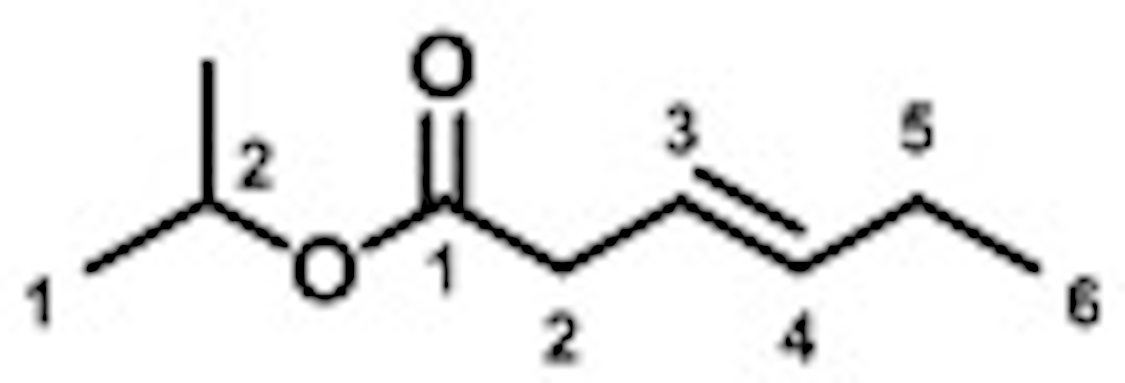

Supplement: S3 Fig — 1H NMR of natural 2-propyl (E)-3-hexenoate: δH (CD2Cl2, 500 MHz) 5.60 (1H, dt, J = 15.4, 6.1 Hz, H-4), 5.51 (1H, dt, J = 15.4, 6.7 Hz, H-3), 5.00 (1H, septet, J = 6.3 Hz, H-2 propyl), 2.98 (2H, dd, J = 6.7, 0.9 Hz, H-2), 2.04 (2H, m, H-5), 1.24 (6H, d, J = 6.3 Hz, H-1 propyl), 0.99 (3H, t, J = 7.5 Hz, H-6); m/z (EI) 156 (M+, 1), 114 (8), 97 (2), 81 (1), 69 (24), 55 (5), 53 (7), 43 (100), 41 (56). (TIFF) [file pone.0206526.s003.tiff]

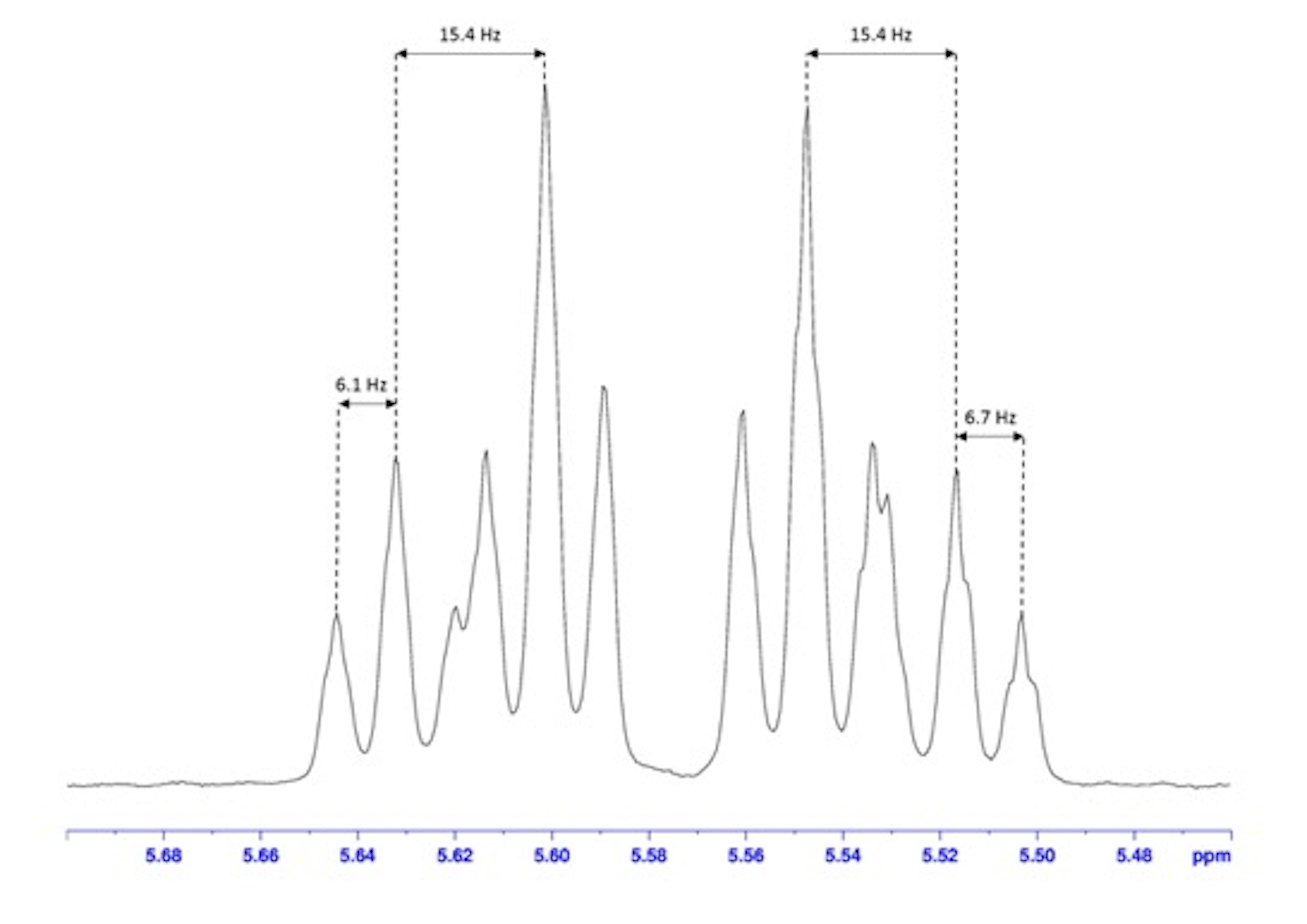

Supplement: S4 Fig — (TIFF) [file pone.0206526.s004.tiff]
