# Supplementary material for: Environmentally vulnerable noble chafers exhibit unusual pheromone-mediated behaviour
Source: PLoS One. 2018 Nov 1;13(11):e0206526. doi: 10.1371/journal.pone.0206526 (PMC6211686; doi:10.1371/journal.pone.0206526)
Supplement: S1 Table — (DOCX) [file pone.0206526.s005.docx]

**S1 Table.** Raw data for olfactometry

Males

| **Stimulus** | Positive response | Negative response | No response |
| --- | --- | --- | --- |
| 1ng 2P3H | 2 | 15 | 0 |
| 10ng 2P3H | 1 | 16 | 0 |
| 100ng 2P3H | 1 | 15 | 1 |
| 1ug 2P3H | 12 | 5 | 0 |
| 10ug 2P3H | 13 | 4 | 0 |
| 100ug 2P3H | 17 | 0 | 0 |
| 2P3H +*H. sphondylium* vs *H. sphondylium* | 14 | 3 | 0 |
| 2P3H +*H. sphondylium* vs 2P3H | 11 | 6 | 0 |
| *H. sphondylium* | 15 | 2 | 0 |

Females

| **Stimulus** | Positive response | Negative response | No response |
| --- | --- | --- | --- |
| 1ng 2P3H | 0 | 11 | 1 |
| 10ng 2P3H | 1 | 11 | 0 |
| 100ng 2P3H | 0 | 12 | 0 |
| 1ug 2P3H | 0 | 12 | 0 |
| 10ug 2P3H | 2 | 10 | 0 |
| 100ug 2P3H | 0 | 9 | 3 |
| 2P3H +*H. sphondylium* vs *H. sphondylium* | 0 | 8 | 4 |
| 2P3H +*H. sphondylium* vs 2P3H | 2 | 0 | 10 |
| *H. sphondylium* | 10 | 0 | 2 |

Analysis script for olfactometry (R)

binom.test(8,8,.5) #opp sex M

binom.test(0,5,.5) #opp sex F

binom.test(8,8,.5) #same sex M

binom.test(0,5,.5) #same sex F

binom.test(2,17,.5) #2p3h 1ng M

binom.test(0,12,.5) #2p3h 1ng F

binom.test(1,17,.5) #2p3h 10ng M

binom.test(1,12,.5) #2p3h 10ng F

binom.test(1,17,.5) #2p3h 100ng M

binom.test(0,12,.5) #2p3h 100ng F

binom.test(12,17,.5) #2p3h 1ug M

binom.test(0,12,.5) #2p3h 1ug F

binom.test(13,17,.5) #2p3h 10ug M

binom.test(2,12,.5) #2p3h 10ug F

binom.test(17,17,.5) #2p3h 100ug M

binom.test(0,12,.5) #2p3h 100ug F

binom.test(15,17,.5) # sphondylium M

binom.test(10,12,.5) #sphondylium F

binom.test(14,17,.5) #2p3h + sphon vs sphon M

binom.test(0,12,.5) #2p3h + sphon vs sphon F

binom.test(11,17,.5) #2p3h + sphon vs 2p3h M

binom.test(2,12,.5) #2p3h + sphon vs 2p3h F
