# Supplementary material for: Environmentally vulnerable noble chafers exhibit unusual pheromone-mediated behaviour
Source: PLoS One. 2018 Nov 1;13(11):e0206526. doi: 10.1371/journal.pone.0206526 (PMC6211686; doi:10.1371/journal.pone.0206526)
Supplement: S2 Table — (DOCX) [file pone.0206526.s006.docx]

**S2 Table.** EAG responses of four antennae from four different male *Gnorimus nobilis* given to six doses of synthetic 2-propyl (*E*)-3-hexenoate. Responses were normalised to those elicited by the control stimulus, 10 µL hexane (=100%), which was applied before and after the dose series.

|  | rep 1 | rep 2 | rep 3 | rep 4 |
| --- | --- | --- | --- | --- |
| Control | 100 | 100 | 100 | 100 |
| 0.01 µg | 105 | 87 | 86 | 98 |
| 0.1 µg | 106 | 115 | 99 | 101 |
| 1 µg | 109 | 116 | 105 | 103 |
| 10 µg | 127 | 129 | 111 | 105 |
| 100 µg | 127 | 132 | 112 | 110 |
| 1000 µg | 123 | 130 | 114 | 105 |
