# Supplementary material for: Environmentally vulnerable noble chafers exhibit unusual pheromone-mediated behaviour
Source: PLoS One. 2018 Nov 1;13(11):e0206526. doi: 10.1371/journal.pone.0206526 (PMC6211686; doi:10.1371/journal.pone.0206526)
Supplement: S3 Table — (DOCX) [file pone.0206526.s007.docx]

**S3 Table.** Field trapping data

trapping_gnorimus.csv

| trap_type | captures | county |
| --- | --- | --- |
| lure | 2 | pershore |
| lure | 2 | pershore |
| lure | 1 | pershore |
| lure | 1 | pershore |
| lure | 2 | pershore |
| lure | 0 | pershore |
| lure | 1 | pershore |
| bloss+lure | 0 | pershore |
| bloss+lure | 2 | pershore |
| bloss+lure | 0 | pershore |
| bloss+lure | 0 | pershore |
| bloss+lure | 2 | pershore |
| bloss+lure | 2 | pershore |
| bloss+lure | 0 | pershore |
| bloss | 0 | pershore |
| bloss | 0 | pershore |
| bloss | 0 | pershore |
| bloss | 0 | pershore |
| bloss | 0 | pershore |
| bloss | 0 | pershore |
| bloss | 0 | pershore |
| contr | 0 | pershore |
| contr | 0 | pershore |
| contr | 0 | pershore |
| contr | 0 | pershore |
| contr | 0 | pershore |
| contr | 0 | pershore |
| contr | 0 | pershore |
| contr | 0 | newFor |
| contr | 0 | newFor |
| contr | 0 | newFor |
| contr | 0 | newFor |
| contr | 0 | newFor |
| contr | 0 | newFor |
| contr | 0 | newFor |
| contr | 0 | newFor |
| contr | 0 | newFor |
| contr | 0 | newFor |
| contr | 0 | newFor |
| contr | 0 | newFor |
| contr | 0 | newFor |
| contr | 0 | newFor |
| contr | 0 | newFor |
| contr | 0 | newFor |
| bloss+lure | 0 | newFor |
| bloss+lure | 0 | newFor |
| bloss+lure | 2 | newFor |
| bloss+lure | 4 | newFor |
| bloss+lure | 3 | newFor |
| bloss+lure | 7 | newFor |
| bloss+lure | 14 | newFor |
| bloss+lure | 6 | newFor |
| lure | 3 | newFor |
| lure | 11 | newFor |
| lure | 8 | newFor |
| lure | 3 | newFor |
| lure | 3 | newFor |
| lure | 4 | newFor |
| lure | 6 | newFor |
| lure | 4 | newFor |

Analysis script (R)

rm(list=ls(all=T)) #clear workspace

#load libraries

library(dunn.test)

library(plyr)

trapping <- read.csv('trapping_gnorimus.csv') #read in file

#england test

dunn.test(trapping$captures[trapping$country=='pershore'],trapping$trap_type[trapping$country=='pershore'],method='bonferroni')

dunn.test(trapping$captures[trapping$country=='newFor'],trapping$trap_type[trapping$country=='newFor'],method='bonferroni')

dunn.test(trapping$captures,trapping$trap_type,method='bonferroni')

tapply(trapping$captures[trapping$country=='pershore'],trapping$trap_type[trapping$country=='pershore'],mean)

tapply(trapping$captures[trapping$country=='newFor'],trapping$trap_type[trapping$country=='newFor'],mean)

tapply(trapping$captures,trapping$trap_type,mean)
